# Supplementary figures and images for: Analysis of the Differences in Volatile Organic Compounds in Different Muscles of Pork by GC-IMS
Source: Molecules. 2023 Feb 11;28(4):1726. doi: 10.3390/molecules28041726 (PMC9961950; doi:10.3390/molecules28041726)

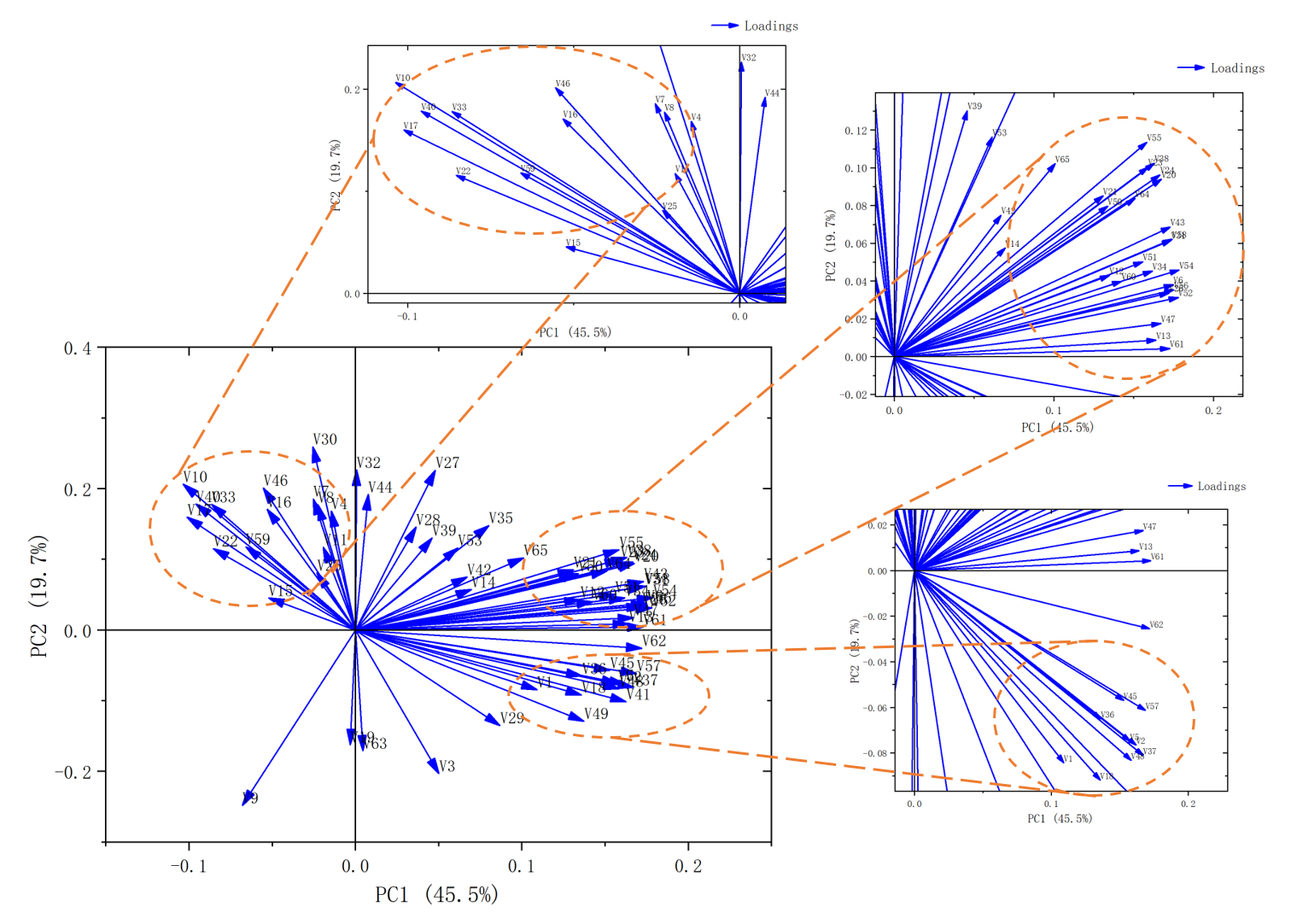

Supplement: Supplementary file 1 [file molecules-28-01726-s001.zip › Figure S1 Loadings plot of PCA.png]
